# Use of Ceftriaxone in Treating Cognitive and Neuronal Deficits Associated With Dementia With Lewy Bodies

**DOI:** 10.3389/fnins.2019.00507

**Published:** 2019-05-24

**Authors:** Ying-Jui Ho, Mei-Shiuan Shen, Chun-Hwei Tai, Hsin-Hua Li, Jian-Horng Chen, Wen-Chieh Liao, Pai-Yi Chiu, I-Yen Lee, Chih-Li Lin, Ching-Sui Hung

**Affiliations:** ^1^Department of Psychology, Chung Shan Medical University Hospital – Chung Shan Medical University, Taichung, Taiwan; ^2^Department of Neurology, College of Medicine, National Taiwan University Hospital – National Taiwan University, Taipei, Taiwan; ^3^Institute of Medicine, Chung Shan Medical University, Taichung, Taiwan; ^4^School of Physical Therapy, Chung Shan Medical University, Taichung, Taiwan; ^5^Department of Anatomy – Department of Pediatrics, Faculty of Medicine, Chung Shan Medical University Hospital – Chung Shan Medical University, Taichung, Taiwan; ^6^Department of Neurology, Show Chwan Memorial Hospital, Changhua, Taiwan; ^7^Division of Urology, Department of Surgery, Tungs’ Taichung MetroHarbor Hospital, Taichung, Taiwan; ^8^Occupational Safety and Health Office, Taipei City Hospital, Taipei, Taiwan

**Keywords:** dementia with Lewy bodies, ceftriaxone, neurogenesis, cognitive dysfunction, neurodegeneration

## Abstract

Dementia with Lewy bodies (DLB) is caused by accumulation of Lewy bodies, destruction of mitochondria, and excess of glutamate in synapses, which eventually leads to excitotoxicity, neurodegeneration, and cognitive impairments. Ceftriaxone (CEF) reduces excitotoxicity by increasing glutamate transporter 1 expression and glutamate reuptake. We investigated whether CEF can prevent cognitive decline and neurological deficits and increase neurogenesis in DLB rats. Male Wistar rats infused with viral vector containing human alpha-synuclein (α-syn) gene, *SNCA*, in the lateral ventricle were used as a rat model of DLB. CEF (100 mg/kg/day, i.p.) was injected in these rats for 27 days. The active avoidance test and object recognition test was performed. Finally, the brains of all the rats were immunohistochemically stained to measure α-syn, neuronal density, and newborn cells in the hippocampus and substantia nigra. The results revealed that DLB rats had learning and object recognition impairments and exhibited cell loss in the nigrostriatal dopaminergic system, and hippocampal CA1, and dentate gyrus (DG). Additionally, DLB rats had fewer newborn cells in the DG and substantia nigra pars reticulata and more α-syn immune-positive cells in the DG. Treatment with CEF improved cognitive function, reduced cell loss, and increased the number of newborn cells in the brain. To our knowledge, this is the first study showing that CEF prevents loss of neurogenesis in the brain of DLB rats. CEF may therefore has clinical potential for treating DLB.

## Introduction

Dementia with Lewy bodies (DLB) is the second most common type of neurodegenerative dementia after Alzheimer’s disease (AD), with a prevalence of up to 5% in the elderly population and approximately 10–25% in the dementia population (McKeith et al., 2005; Zaccai et al., 2005). Clinically, patients with DLB have cognitive and motor symptoms that sometimes overlap with those of AD and Parkinson’s disease (PD) (Mueller et al., 2017). DLB is characterized by fluctuations in cognition and alertness, recurrent visual hallucinations, parkinsonism, and rapid eye movement sleep behavior disorders; the presence of two of these symptoms is sufficient for diagnosis of probable DLB (McKeith et al., 2005; Kemp et al., 2017). Other supportive clinical features, which are less specific, are severe neuroleptic sensitivity, repeated falls, autonomic dysfunction, anxiety, and depression (Kemp et al., 2017). Currently, DLB has no cure. Therefore, identifying a more effective medication has become crucial.

Pathologically, the cardinal feature of DLB is the widespread accumulation of intracytoplasmic Lewy bodies (Oda et al., 2009; Macijauskienė and Lesauskaitė, 2012), first described by Friedrich Henrich Lewy in 1912. Lewy observed large eosinophilic spherical or kidney-shaped intracytoplasmic inclusions abundantly expressed in the cortical/subcortical neuronal bodies in patients with paralysis agitans, but did not provide much clinical detail (Weil et al., 2017). In Kosaka et al. (1976) reported an autopsy case of unclassifiable presenile dementia, which evidenced the association between Lewy bodies and dementia; the unclassifiable dementia was subsequently termed DLB. Immunohistochemical techniques and subsequent use of anti-α-synuclein antibodies (Spillantini et al., 1997) further revealed that the primary component of Lewy bodies is α-synuclein (α-syn) in DLB (Spillantini et al., 1998; Beyer et al., 2009; Kim et al., 2014). α-Syn is a 140-amino soluble protein encoded by *SNCA*, which is abundantly expressed in the central nervous system (Spillantini et al., 1998; Beyer et al., 2009; Kim et al., 2014). Genetic mutations in α-syn, including point mutations (A53T, A30P, and E46K) and multiplications, have been linked to the neuropathology of DLB (Singleton et al., 2003; Zarranz et al., 2004). However, the exact mechanism of α-syn aggregation and the formation of Lewy bodies as well as the mechanism by which it induces toxicity to neurons are still unclear. Although the presence of a high degree of neuronal death at sites of misfolded α-syn aggregation suggested the possible involvement of aggregated α-syn inclusions in the cytotoxicity in DLB (Valdinocci et al., 2017), overexpression of wild-type α-syn itself do not cause α-syn aggregation (Ko et al., 2008). Moreover, no pathogenic alterations or polymorphism in *SNCA* was found in DLB, indicating that mutated α-syn does not play a major role in DLB (Tagliafierro and Chiba-Falek, 2016). Therefore, some additional factors may contribute to the abnormal α-syn aggregation in DLB.

Although α-syn is the main component of Lewy bodies in DLB, it is originally found in the senile plaques of the brains of patients with AD. Up to 50% of familial and sporadic patients with AD exhibit Lewy bodies upon autopsy. Cortical deposition of beta-amyloid (Aβ) and neurofibrillary tangles, both of which are known to be involved in AD neuropathogenesis, are also frequently observed in individuals with DLB (Hishikawa et al., 2003) and are linked to disease severity (Armstrong and Cairns, 2009). Aβ accelerates the oligomerization of α-syn, which accentuates its toxicity and leads to neural and behavioral deterioration caused by α-syn (Overk et al., 2014). Moreover, direct or indirect interactions between α-syn and Aβ promote their mutual aggregation and accumulation, disturb the function of mitochondria, cause excessive glutamate release in synapses, and eventually result in excitotoxicity and cell death in the cortex, striatum, and limbic system. Evidence indicates that Aβ may elicit oxidative and inflammatory reactions and suppress the clearance of α-syn, which directly promotes α-syn protein accumulation, thereby triggering the pathological effects of α-syn (Marsh and Blurton-Jones, 2012; Lin et al., 2016). In other words, Aβ aggravates the neurotoxicity of α-syn in the brain (Lin et al., 2016).

Viral vectors containing human α-syn gene, *SNCA*, can constantly express α-syn proteins in a host. Some studies have injected vectors to deliver *SNCA* into the hippocampus, cortex, and striatum of rats and used α-syn expression to indicate the disease state of DLB (Lim et al., 2011; Overk et al., 2014). The use of viral vectors for *in vivo* gene transfer has many advantages, namely low cost, fast transduction, and high expression of transferred genes. Additionally, specific brain regions of interest (ROIs) can be targeted (Mochizuki et al., 2006). Although viral vectors can deliver specific genes to the brain ROIs of the host, the transduction essentially remains confined to the area near the injection site. Therefore, more widespread transduction in larger brain regions such as the cortex remains challenging (Chesselet, 2008; Aldrin-Kirk et al., 2014). In the present study, we reidentified the major hallmarks of DLB by directly injecting α-syn gene vector into the lateral ventricle of rat brains. The rationale behind this viral-based genetic rat model was that, because viral vectors are diffused widely with the flow of cerebrospinal fluid, *SNCA* may be transferred and α-syn expression can be induced in widespread brain regions. We evaluated the validity of this animal model of DLB by examining its reproducibility in both neuronal and cognitive symptoms (e.g., changes in learning and memory capability) of the disease.

Currently, no specific medicine is available for treating DLB. Because DLB and AD share many clinical features, which implies common underlying pathophysiology (Samuel et al., 1997), the pharmacological therapy strategies for AD, such as cholinesterase inhibitors and *N*-methyl-D-aspartate glutamate receptor antagonists (e.g., memantine), are used to alleviate DLB symptoms. Although these drugs have the potential to alleviate cognition symptoms (van Marum, 2009), numerous adverse side effects such as hallucinations and schizophrenia-type symptoms have limited tolerability to these drugs (Aarsland et al., 2009; Wang et al., 2015). Recent clinical and preclinical evidence has led to identification of a new drug target. By screening more than 1000 FDA-approved drugs, Rothstein and his colleagues found that a beta-lactam antibiotic, ceftriaxone (CEF), can increase expression of astrocytic glutamate transporter-1 (GLT-1) through transcriptional activation and investigated its biochemical and functional activities *in vivo* (Rothstein et al., 2005). Thus, CEF is considered to have the potential for enhancing glutamate clearance in the synaptic cleft, thereby preventing the excitotoxicity that contributes to neuronal injury and death in neurodegenerative diseases (Rothstein et al., 2005). Our previous study demonstrated that CEF ameliorated behavioral and neuronal deficits and increased neurogenesis in the hippocampus and substantia nigra in the 1-methyl-4-phenyl-1,2,3,6-tetrahydropyridine (MPTP)-induced PD rat model (Hsu et al., 2015). Because glutamatergic hyperactivity and excitotoxicity participate in the pathophysiology of DLB, we also evaluated the efficacy of the novel therapeutic agent CEF in improving neurological and behavioral deficits in the DLB rat model in this study.

## Materials and Methods

### Animals

Twelve-week-old male Wistar rats (weighting: 420 ± 30 g; *n* = 25; BioLASCO Taiwan Co., Ltd., China) were randomly assigned to groups of 3–4 and housed in acrylic cages (35 cm × 56 cm × 19 cm) in a temperature-controlled animal room (21∼25°C) with free access to food and water. Photoperiods in rat rooms were controlled by an automatic timer to provide 12 h of light (from 7:00 to 19:00 h) and 12 h of dark (from 19:00 to 7:00 h) cycle. In order to minimize defensive behaviors and stress responses to the experimenter, prior to the start of the experiment all animals were handled for 5 min/day for two consecutive days. All behavioral observations were conducted during the light phase and the animals were transferred to the observation room under dim illumination (red light of 28 lx) with 70 dB of white-noise masking to reduce the ambient environmental interference to animals. The instrument was cleaned with 20% ethanol before the experiment. Before the conductance of behavioral tests, the animals were allowed to adapt for 15 min in the observation room and to be familiar with the environment. All experimental procedures were performed according to the NIH Guide for the Care and Use of Laboratory Animals and were approved by the Animal Care Committee of Chung Shan Medical University (IACUC approval No. 1455). All efforts were made to minimize animal suffering and to reduce the number of animals used (Castelhano-Carlos and Baumans, 2009).

### General Procedures

The rats were randomly divided into three groups, i.e., “Sham+saline” (*n* = 10), “DLB+saline” (*n* = 7) and “DLB+CEF100” groups (*n* = 8), and underwent stereotaxic brain surgery on day 0 as described in our previous reports (Castelhano-Carlos and Baumans, 2009). Briefly, all rats were anesthetized by i.p., injection of Zoletil (20 mg/kg; Virbac, Carros, France) and mounted in a stereotaxic frame. For rats in the “DLB+saline” and “DLB+CEF100” groups, recombinant adeno-associated viral (rAAV) vector containing human α-syn gene, *SNCA*, (10 μl) were injected into the left lateral ventricle to induce whole-brain α-syn overexpression using the following coordinates adapted from the rat brain atlas (AP: -0.8 mm, ML: -1.5 mm, DV: -3.6 mm from the bregma, midline and skull surface, respectively). In addition, 5 μl of Aβ_1-42_ (10 μg) solution was bilaterally infused in their prefrontal cortex using the following stereotaxic coordinates (AP: 1.6 mm, ML: ± 2.0 mm, DV: -2 mm from the bregma, midline, and skull surface, respectively). Aβ peptide (Anaspec, United States) was prepared as described previously (Kornelius et al., 2015). Briefly, Aβ lyophilizates were dissolved at 10 mM in 10% 60 mM NaOH and 90% 10 mM phosphate buffer (pH 7.4) as an alkaline stock reagent. The stock solution was then diluted to 100 μM Aβ in 50 mM phosphate buffer, 150 mM NaCl, pH 7.4, and incubated for 24 h at 4°C to obtain Aβ oligomers. The human wild-type α-syn coding sequence was amplified and cloned into the SPORT6-pCMV or rAAV vectors. For *in vivo* transfection, rats were stereotaxically injected with rAAV-pCMV-SNCA (viral titer: 1.5E12 genome copies per mL). Production of rAAV vector constructs was based on previous report (Aldrin-Kirk et al., 2014). On the contrary, rats in the “Sham+saline” group were infused with 10 μl and 5 μl of saline, respectively, in the left lateral cerebral ventricle and bilateral prefrontal cortex with the stereotaxic coordinates mentioned above. During the five post-operative days, the rats were housed individually in plastic cages and 10% sucrose solution was provided *ad libitum* to prevent weight loss after surgery and reduce mortality.

Ceftriaxone or saline (control) treatment started from day 1 post-operation and continued for 27 days. Animals in the “Sham+saline” and “DLB+saline” groups received saline injection (1 ml/kg/day, i.p.) while animals in the “DLB+CEF100” group received CEF treatment (100 mg/kg/day, i.p.) (Roche, Kaiseraugst, Switzerland). The rationale of using the dosage (100 mg/kg/day) of CEF in the present study was based on our previous studies, where the treatment of CEF at the dosage restored working memory and object recognition in the MPTP-induced PD rat model (Hsu et al., 2015), Furthermore, the treatment of CEF at the dosage of 200 mg/kg/day for 5 days suppressed transient forebrain ischemia-induced loss of GLT-1 and have protective effects on CA1 neurons (Ouyang et al., 2007).

Cognitive functions were assessed with two behavioral tests, namely the object recognition test on day 25∼27 and the active avoidance test on day 26.

In addition, in order to examine treatment effect on neurogenesis in the dentate gyrus (DG) and in the substantia nigra (SN), all animals received injection of 5′-bromo-2′-deoxyuridine (BrdU, 150 mg/kg, i.p.), a marker of proliferating cells, dissolved in saline at a concentration of 25 mg/ml, at 17:00 and 23:00 h on day 27. After the experimental procedure, all rats were euthanized (on day 28) by exposure to CO_2_ and transcardially perfused with phosphate-buffered saline (PBS) with their brains fixed using 4% paraformaldehyde immediately before being removed. The fixed brains were post-fixed and dehydrated in 40% sucrose solution containing 4% paraformaldehyde.

### Behavioral Tests

#### Object Recognition Test

**FIGURE 1:**
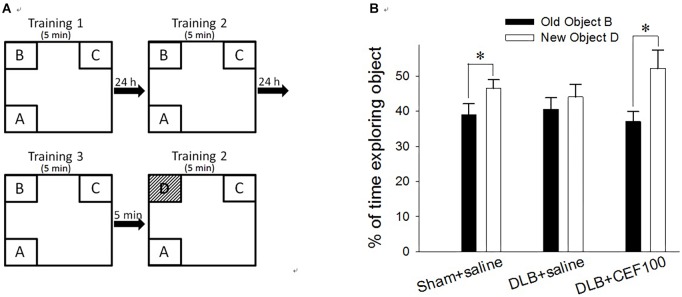
Effect of ceftriaxone (CEF) on behavioral performance in the object recognition test. **(A)** Schematic diagram of the schedule and arrangement of the objects in the object recognition test. **(B)** Rats were infused with Aβ in the prefrontal cortex and with recombinant adeno-associated viral (rAAV) vector containing *SNCA* in the lateral ventricle to model DLB but in sham-operated rats, these brain regions were infused with saline instead. CEF or saline (control) treatment started from day 1 post-operation and continued for 27 days. The sham-operated rats received only saline injection (1 ml/kg/day, i.p., “Sham+saline” group) while DLB rats were sub-divided into two groups, receiving either CEF (100 mg/kg/day, i.p., “DLB+CEF100” group) or saline (1 ml/kg/day, i.p., “DLB+saline” group) injection. ^∗^*P* < 0.05, compared with the percentage of time spent on exploring object B. The data are expressed as the mean ± SEM.

Recognition ability was measured using the object recognition test. Rats have a natural tendency to spend more time exploring novel objects than familiar ones in the same context. This novelty preference provides an index of object recognition by measuring whether the rats can distinguish between new objects and old objects. The apparatus, an open box (60 cm long × 60 cm wide × 60 cm high) was divided into nine areas, and three out of the four objects (A, B, C, or D) were fixed on the floor in three corners of the arena while the rat was placed in the other corner of the open box (Figure 1A). No further changes were made once the location had been determined. All objects are the same during the training period, but the object B was replaced by a novel object D during the test period.

Each rat was subjected to three training sessions separated at 24 h interval. In the training session, the rat was allowed to explore the objects in the open box for 5 min; then, 5 min after the last training session, a test session was performed. In the test session, the object “B” was replaced by a novel object, “D,” while, following object replacement, the animal returned to explore the open box for another 5 min. The exploration time spent on the object B in the training session and on the object D in the test session was calculated in percentage of the time spent exploring objects [(time _BorD_/time _allobjects_) × 100%]. The difference in the percentage of time spent exploring the old object “B” and the novel object “D” served as a measure of recognition ability for the familiar object.

#### Active Avoidance Test

**FIGURE 2:**
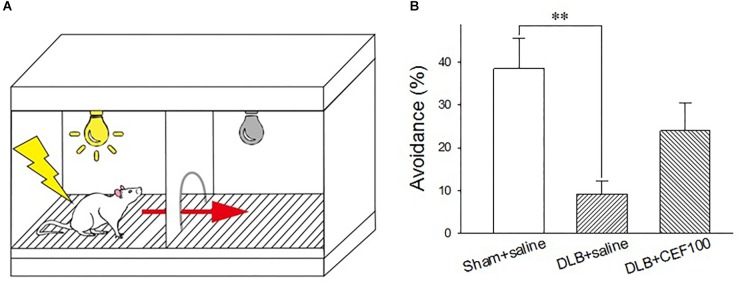
Effect of ceftriaxone (CEF) on the percentage of avoidance in the active avoidance test. **(A)** Schematic representation of the shuttle box used in the active avoidance test. **(B)** The treatment and group name are the same as those in Figure 1. DLB+saline group had a lower percentage of avoidance, compared with the Sham+saline group. *^∗∗^P* < 0.01, compared with Sham+saline group. The data are expressed as the mean ± SEM.

The active avoidance test was used to assess the fear-based conditioned avoidance learning that is an operational conditioning as well. The behavior was tested in a two-compartment shuttle box (AccuScan Instruments, Inc., AI0506SHF512R, United States), consisting of two equal-size compartments (22.5 cm long × 21.5 cm wide × 33 cm high) that had an electrified grid floor made up of stainless steel bars and shared a central door (diameter 7.5 cm). The central door was opened to allow the animals to cross to the opposite chamber. The grid floor had parallel stainless steel bars spaced 0.7 cm apart, which can deliver electric shock (Figure 2A).

Rats learned to pair a conditioned stimulus (CS, 115 db tone plus light, 3 s) with an aversive unconditioned stimulus (UCS, 0.5 mA electric foot shock, a maxima of 10 s) and to prompt a correct response (e.g., moving to the safe chamber of the shuttle box). The test started with a 60-s free exploration of the shuttle box where it did not matter in which chamber the rat was placed initially. Then a total of 25 trials were carried out with the inter-trial interval of 47–57 s. In each trial, the CS came up in the chamber where the rat was. If the rat crossed the central door during the presence of the CS, no UCS will be delivered. If rats failed to act during the 3 s of CS, the UCS would be delivered together with the presence of CS. Delivery of CS and UCS were controlled by the software, ShuttleFlex System. Three dependent variables were measured: active avoidance response (correct move during the CS presence), escape response (move following onset of UCS), and escape failure (failure to perform a move during the UCS).

### Histological Assessment

For immunostaining, the brain tissues were rapidly frozen and sectioned (25 μm thick) in the coronal plane through the brain on a cryostat. Serial sections containing three regions of interest (ROIs), including striatum (bregma 2.04 to 0.96 mm), hippocampus (bregma –2.76 to–4.20 mm) and substantia nigra (SN, bregma –4.80 mm to –5.76 mm), were collected referring to the rat brain atlas (Paxinos and Watson, 1986), mounted on gelatinized slides and kept in PBS until being immunostained. The immunostaining assays used for histological analysis were detailed below, which were used to detect: (1) the density of the pyramidal neurons in the hippocampal dentate gyrus (DG); (2) the density of dopaminergic (DAergic) neurons and terminals in the SN pars compacta (SNc) and in the striatum, respectively; and (3) the neurogenesis in the hippocampal DG and in the SN.

#### Nissl Staining

The Nissl staining was used to detect the neuronal density in the hippocampus. A series of adjacent sections containing hippocampus were washed twice for 15 min in 0.01 M PBS and incubated in 0.5% cresyl violet staining solution (Hsieh et al., 2012a; Hsu et al., 2015; Weng et al., 2016).

#### Tyrosine Hydroxylase (TH) Staining

Immunohistochemical staining of TH was used to evaluate DAergic neurons in the SNc and DAergic terminals in the striatum, respectively. The TH staining was performed as described in our previous reports (Hsieh et al., 2012b; Huang et al., 2015; Weng et al., 2016). Briefly, frozen brain sections containing the striatum and SNc were immunostained with rabbit polyclonal antibodies against rat TH (1:500 dilution in PBS; Millipore, CA, United States) at room temperature overnight. Then, sections were sequentially incubated with biotinylated horse anti-rabbit IgG antibodies (1:300 dilution in PBS; Vector Laboratory, CA, United States) at 37°C for 1 h, followed by incubating with streptavidin-horseradish peroxidase (1:300 dilution in PBS; Biorad Laboratories, Oxford, United Kingdom) at 37°C for 30 min, and with 0.02% 3,3′-diaminobenzidine tetrachloride (DAB) (Sigma, United States) at room temperature for 30 min. After that, the slides were extensively washed with PBS.

#### Neurogenesis (Bromodeoxyuridine, BrdU) Staining

Bromodeoxyuridine staining was used to detect newborn cells in the hippocampal DG and in the SN. For immunostaining the BrdU-labeled cells, the coronal sections containing the hippocampus and SN were rinsed (3 × 5 min each) with 0.05 M TB, incubated for 20 min at room temperature with 0.3% hydrogen peroxide, reacted with 0.5% Triton-100 for 15 min to increase permeability of cells, denatured by incubation for 30 min at 37°C with 2N HCl, and blocked by incubation for 1 h at room temperature in 100% normal goat serum dissolved in TB containing 0.5% Triton-100. They were then incubated overnight at 4°C with mouse monoclonal antibody against BrdU (1:100; Cell Signal, United States) and incubated sequentially for 1 h at room temperature with biotinylated horse anti-mouse IgG (H+L) antibodies (1:200; Vector, United States), 30 min at 37°C with streptavidin-horseradish peroxidase (1:300), and 40 s at room temperature with 3,3′-diaminobenzidine tetrachloride (DAB, Sigma, United States), then dehydrated in ethanol and xylene and coverslipped.

#### Image Analysis

After immunostaining, images of histological sections were captured using a microscope (ZEISS AXio Imager A2, Germany) coupled to a CCD (Optronics, United States). The contour delineations of the area of interest (AOI were defined according to the rat brain atlas (Paxinos and Watson, 1986) and were drawn with the Q Capture Pro 7 software (Media Cybernetics, CA, United States). The cell number was counted by the Image Pro Plus Software 6.0 (Media Cybernetics, CA, United States).

The counting of the pyramidal cells in the hippocampal DG and CA1 region was performed as published in our previous reports (Hsieh et al., 2012b; Hsu et al., 2015; Weng et al., 2016). The following parameters were calculated: optical density (OD) of TH immunoreactivity in the striatum; densities of DAergic neuron in the SNc and pyramidal cell (percentage of neuronal area) in the hippocampus, and neuronal area in the DG. The images of α-syn immune-positive cells and BrdU labeled cells were acquired using a microscope (ZEISS AXioskop2, Germany) and the density of cells was counted.

To determine the number of BrdU-labeled cells in the DG and in the SN, we use the semi-quantitative method, a modified version of the fractionator principle, that was based on a stereological method reported in the literature (Kronenberg et al., 2003; Zhang et al., 2013; Hsieh et al., 2017). The thickness of the DG is 1,360 μm (4,160–2,800 μm), thus the DG produced 54 sections (1360/25 μm). The thickness of the SNc is 900 um (6,040–5,140 μm), thus the SN produced 36 sections (900/25 μm). We stained a one-in-six series of sections covering the entire hippocampus and SN in its rostrocaudal extension. BrdU labeled cells were counted exhaustively in the SN and in the granule cell layer and the subgranular zone (SGZ) of DG. The total number of BrdU-labeled cells was then estimated by multiplying the resulting counts by six because every sixth section had been used. The numbers obtained in our study are thus absolute numbers per SN and per DG.

### Data Analysis

SPSS 17.0 statistical software was used for data analysis. Analysis of variance (ANOVA), followed by the least-significant difference (LSD) *post hoc* test, was used to analyze the active avoidance test and histological results, while the paired-samples *t*-test were used to analyze the object recognition test data. All results are expressed as the mean ± SEM. The level of significance was defined as *P* < 0.05.

## Results

### Behavioral Tests

#### Object Recognition Test

Paired-sample *t*-test was used to measure the differences between the percentage of exploration time the rat spent on old object “B” and that on novel object “D” for each group to check CEF restoring animal’s object recognition ability in the object recognition test. Both control (Sham+saline) and DLB rats treated with CEF at the dosage of 100 mg/kg/day (DLB+CEF100) spent a significantly higher percentage of time exploring novel object “D” than exploring old object “B” (df = 6, Sham+saline group: *t* = –2.52, DLB+CEF100 group: *t* = –3.62, both *P*-values < 0.05). However, no difference in the percentage of the time exploring objects “B” and “D” was observed in the DLB+saline group (df = 6, *t* = –0.91, *P* = 0.40), indicating an impairment in object recognition (Figure 1B).

#### Active Avoidance Test

One-way ANOVA revealed a group difference in the percentage of avoidance (correct moves during the CS) in the active avoidance test [*F*(2,21) = 6.14, *P* < 0.01]. The LSD *post hoc* analysis showed that the main difference occurred between “Sham+saline” and “DLB+saline” groups where the percentage of avoidance was significantly lower in the “DLB+saline” group (9 ± 3%) than the “Sham+saline” group (38 ± 7%) (*P* < 0.01). CEF treatment (“DLB+CEF100” group, 24 ± 6%) could slightly protect the DLB rats against task performance deficit. However, we may need more conservative inferences because no statistic differences were observed between the DLB+saline and DLB+CEF groups (Figure 2B).

### Histological Assay

#### Density of Pyramidal Neurons in the Dentate Gyrus (DG)

**FIGURE 3:**
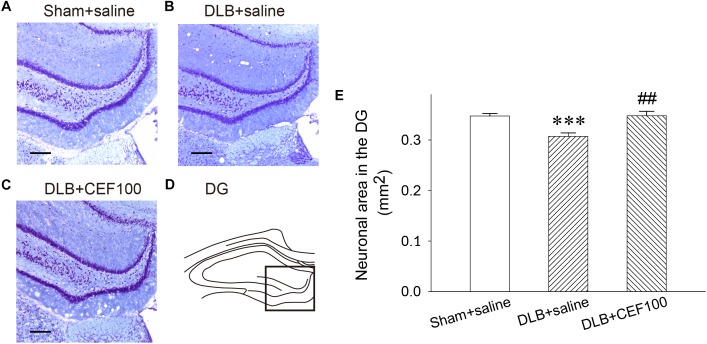
Effect of ceftriaxone (CEF) on neuronal density in the hippocampal dentate gyrus (DG). The treatment and group name are the same as those in Figure 1. **(A–C)** Nissl-stained pyramidal cells in the DG of the hippocampus are demonstrated by the representative coronal section of each group. Magnification, 100×; bar, 100 μm. The square in **(D)** indicates the area used for measuring the density of the Nissl-stained pyramidal neurons in the DG region of the hippocampus. **(E)** Quantitative results. *^∗∗∗^P* < 0.001, compared to Sham+saline group; *^##^P* < 0.01, compared to DLB+saline group. The data are expressed as the mean ± SEM.

In the hippocampal DG, one-way ANOVA revealed that density of Nissl-stained pyramidal neurons had a significant group difference [*F*(2,14) = 10.58, *P* = 0.02]. The LSD *post hoc* test indicated that the density of the DG neurons was significantly lower in the DLB+saline group as compared with the Sham+saline group (*P* = 0.001) and with the DLB+CEF100 group (*P* < 0.01). However, compatible DG neuronal density was observed between the Sham+saline and the DLB+CEF100 groups (Figure 3). This result suggested that there was a neuronal loss in the hippocampal DG of the DLB rats and this could be protected by CEF treatment.

#### Density of DAergic Terminals in the Striatum

**FIGURE 4:**
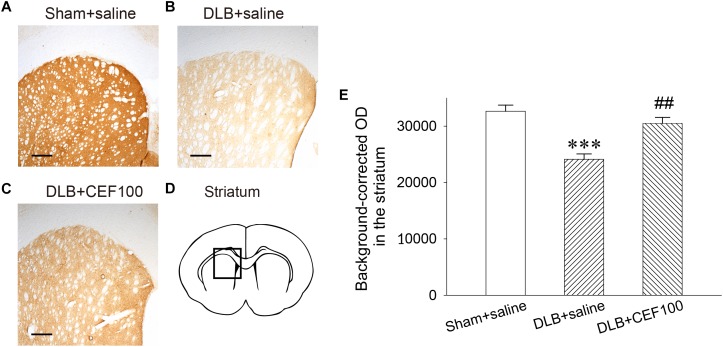
Effect of ceftriaxone (CEF) on tyrosine hydroxylase immunoreactivity in the DAergic terminals of the striatum. The treatment and group name are the same as those in Figure 1. **(A–C)** Tyrosine hydroxylase immunoreactivity in the DAergic terminals of the striatum is demonstrated by the representative coronal section of each group. Magnification, 50×; bar, 200 μm. The square in **(D)** indicates the area where the TH immunoreactivity was measured. **(E)** Quantitative results. *^∗∗∗^P* < 0.001, compared with Sham+saline group; *^##^P* < 0.01, compared with DLB+saline group. The data are expressed as the mean ± SEM.

Tyrosine hydroxylase immunoreactivity was used to measure the density of DAergic terminals in the striatum (Figure 4). One-way ANOVA revealed that the TH immunoreactivity at the DAergic terminals of the striatum showed a significant group difference [*F*(2,14) = 15.83, *P* < 0.001]. The LSD *post hoc* analysis indicated that rats in the DLB+saline group significantly reduced 13% of TH immunoreactivity in the striatal DAergic system compared with both Sham+saline group (*P* < 0.001) and DLB+CEF100 group (*P* < 0.01) while the comparison between the latter two groups showed no significant difference. This result indicated that striatal DAergic loss occurred in the current DLB rat model and CEF treatment restored density of DAergic terminals in the striatum (Figure 4).

#### Density of DAergic Neurons in the Substantia Nigra Pars Compacta (SNc)

**FIGURE 5:**
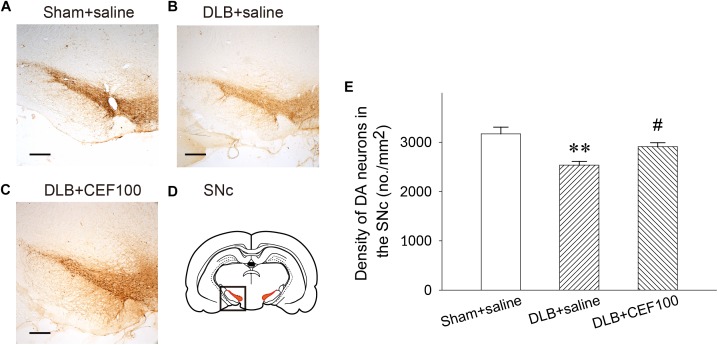
Effect of ceftriaxone (CEF) on density of DAergic neurons in the substantia nigra pars compacta SNc. The treatment and group name are the same as those in Figure 1. **(A–C)** The density of DAergic neurons in the SNc is demonstrated by the representative coronal section of each group. Magnification, 50×; bar, 200 μm. The square in **(D)** indicates the area where the neuronal density of DAergic neurons was measured. **(E)** Quantitative results. *^∗∗^P* < 0.01, compared with Sham+saline group; *^#^P* < 0.05, compared with DLB+saline group. The data are expressed as the mean ± SEM.

In the SNc, one-way ANOVA revealed that the density of DAergic neurons in the SNc showed a significant group difference [*F*(2,14) = 7.74, *P* < 0.01]. LSD *post hoc* analysis showed that rats in the DLB+saline group had a significant lower density of DAergic neurons in the SNc when compared with the Sham+saline group (*P* < 0.01) and the DLB+CEF100 group (*P* < 0.05) while the density of DAergic neurons are compatible in the latter two groups (Figure 5).

#### Neurogenesis in the DG and SN

**FIGURE 6:**
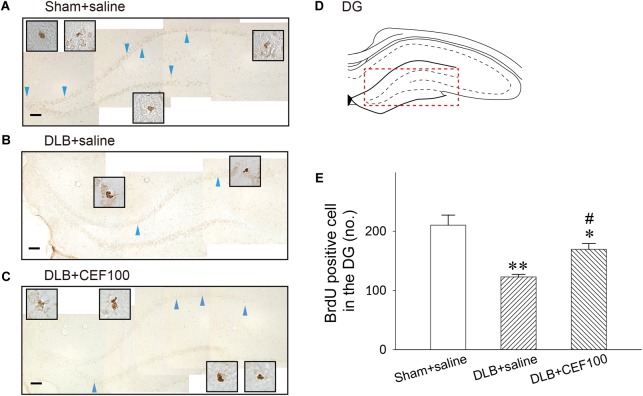
Effect of ceftriaxone (CEF) on neurogenesis in the hippocampal dentate gyrus (DG). The treatment and group name are the same as those in Figure 1. **(A–C)** Newly generated cells are indicated by BrdU labeling in the representative coronal section of each group. Magnification, 200×; bar, 50 μm. High magnification images (1000×) of BrdU-positive cells are shown in the insets. **(D)** DG. **(E)** Quantitative results. *^∗^P* < 0.05; *^∗∗^P* < 0.01, compared with Sham+saline group. *^#^P* < 0.05, compared with DLB+saline group. The data are expressed as the mean ± SEM.

**FIGURE 7:**
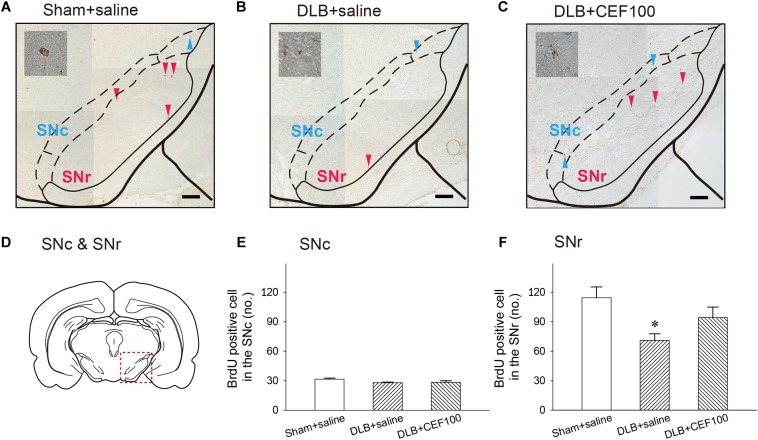
Effect of ceftriaxone (CEF) on neurogenesis in the substantia nigra (SN). The treatment and group name are the same as those in Figure 1. **(A–C)** Coronal sections in the SN. Newborn cells are indicated by BrdU labeling in representative coronal sections. Magnification, 200×; bar, 50 μm. High magnification images (1000×) of BrdU-positive cells are shown in the insets. **(D)** SN. Quantitative results of pars compacta (SNc) **(E)** and of pars reticulate (SNr) **(F)** are shown. *^∗^P* < 0.05, compared with Sham+saline group. The data are expressed as the mean ± SEM.

The cell proliferation, newborn cell, was assayed by BrdU labeling in the hippocampal DG (Figure 6) and SN (Figure 7). In the hippocampal DG, one-way ANOVA revealed that there was a significant group difference [*F*(2,14) = 10.05, *P* = 0.03]. *Post hoc* analysis using the LSD test indicated that the BrdU-positive cell number of the DLB+saline group (122 ± 8) was significantly lower comparing either with the Sham+saline group (210 ± 41, *P* < 0.01) or with DLB+CEF100 group (169 ± 21, *P* < 0.05).

The ANOVA revealed that number of BrdU-positive cell in the SNc showed no between-group difference [*F*(2,14) = 2.47, *P* = 0.127]. While in the substantia nigra pars reticulata (SNr), the number of BrdU-positive cell showed a significant-group difference [*F*(2,14) = 4.15, *P* = 0.04], and *post hoc* analysis showed that rats in DLB+saline group (71 ± 13) had a lower number of BrdU-positive cell, compared with Sham+saline group (114 ± 27) (*P* < 0.05). DLB+CEF100 group (94 ± 23) showed no difference in the number of BrdU-positive cell, compared with Sham+saline group (Figure 7).

## Discussion

The present study revealed that DLB rats exhibited neuronal loss, cognitive dysfunction, and reduced neurogenesis. CEF treatment improved cognitive decline and neurological deficits and restored neurogenesis in the DLB rats. Thus, CEF may have clinical potential for prevention and treatment of DLB.

Lewy bodies and Lewy neurites formed by α-syn accumulation are involved in DLB pathophysiology (Campbell et al., 2000; Schulz-Schaeffer, 2010) and result in neurotoxicity and cell loss in the brain (Kim et al., 2014; Burre et al., 2015; Ingelsson, 2016). Interactions between α-syn and Aβ promote α-syn aggregation (Lin et al., 2016). Thus, in addition to injecting the α-syn gene vector into the lateral ventricle of rats, Aβ was injected into the bilateral prefrontal cortex in the present study to accurately model the pathological characteristics of DLB.

Aldrin-Kirk et al. (2014) reported a first rat model of widespread progressive synucleinopathy throughout the forebrain using local injection of adeno-associated viral (AAV) vector expressing human α-syn into the striatum of rats. The local injection of AAV induced a wide transport of α-syn throughout the brain (Aldrin-Kirk et al., 2014). Thus, it is no need to inject AAV in a certain brain area for inducing expression of α-syn at the area. But the striatal injection of AAV vector caused a prominent α-syn expression in the cortex and a lesser extent in the limbic area, the mechanism underlies which may be the anterograde transport (Aldrin-Kirk et al., 2014). Hippocampus, the brain structure surrounding the caudal parts of the ventricles, is involved in cognitive function and plays an important role in pathological changes in DLB. Hence for inducing a transduction of α-syn in the hippocampus, injection of vector into the ventricle was selected in the present study, by which the vector perfused in the CSF and can approach all the brain areas. Our previous *in vitro* study has demonstrated that Aβ treatment in α-syn-overexpressed neurons significantly increased intracellular α-syn aggregation and cytotoxicity, indicating the interaction of these two proteins (Chang et al., 2018). Considering AAV particles diffuse very efficiently in the CSF, we recently injected rAAV into the ventricle and Aβ into the hippocampus of rats. We found that α-syn overexpressed in all the brain areas and that Aβ inhibited autophagy and enhanced α-syn aggregation and cell loss in the hippocampus (Lin et al., 2016). To mimic the pathological changes of DLB, Aβ was injected into the cortex in the present study because of that Aβ affects nearby areas and that up to one-fourth of DLB patients shows cortical deposition of Aβ (Hishikawa et al., 2003). Moreover, it is worthy to elucidate how Aβ in the cortex affects α-syn accumulation in the hippocampus and to analyze correlation of the two proteins. For avoiding local high concentration and unequal distribution, rAAV was not injected into a certain brain area. For avoiding high volume damage, rAAV and Aβ were not injected at a same brain area. Consistent to our previous report, we observed α-syn accumulation and cell loss in the hippocampus (Ho et al., 2018). These neurohistological changes were related to the behavioral defects in learning and recognition, which is consistent with literature reports showing that α-syn is involved in neurodegenerative synucleinopathies and in the onset and progression of DLB (Galvin et al., 1999).

In accordance with reports in the literature showing robustly expression of α-syn mRNA in forebrain, including striatum, motor cortex, frontal cortex, olfactory bulb, and substantia nigra, 10 months after AAV injection in neonatal rat (Aldrin-Kirk et al., 2014), our unpublished data showed a 12-fold higher level of α-syn mRNA expression 6 weeks after AAV injection. The increase of α-syn protein level was compatible to the mRNA enhancement. However, analyzing time course of α-syn mRNA expression and correlation between the expression of α-syn and the increase of α-syn immuno-positive cells will provide insight into the role of α-syn accumulation in pathological changes in DLB.

Synucleinopathies share a number of features in pathology of neurological disorders. α-Syn and Aβ cause neuroinflammation and neurodegeneration, which have been the center of focus in understanding the etiology of these disorders (Kim et al., 2014). We reported a method of injection of Aβ and viral vectors with the *SNCA* gene into the brain to induce the DLB rat model (Lin et al., 2016). Consistent with our report (Ho et al., 2018), the present study revealed that DLB rats showed a high level of α-syn accumulation in the hippocampal DG and a lower density of pyramidal neurons in the hippocampal CA1 and DG.

It has been reported that severity of cognitive impairment in DLB is correlated with the density of Lewy neurites in the hippocampus, raising the possibility that disruption of the connection between the dentate gyrus, entorhinal cortex, septal nuclei, and hypothalamus and the CA1 contributes to dementia (Churchyard and Lees, 1997). The evaluation of α-syn accumulation and neuronal density in the hippocampal DG supported the above findings. Analyzing the cortical density of Aβ in the future study will provide further information for speculating the role of Aβ in the pathophysiology.

Recognition memory refers to the ability to recognize previously encountered events, objects, or places. When a past event is re-experienced, the animal compares the environmental stimuli with stored memories, elicits matching signals, and produces responses (Norman and O’reilly, 2003). Patients with DLB have visual construction and perception defects. Even in a familiar street or at home, patients may lose their sense of direction and become lost (Mosimann et al., 2004). A study evaluated the performance of patients with DLB in a delayed matching-to-sample test (a test used to evaluate visual recognition memory), where, compared with healthy controls, patients with DLB obtained a lower visual recognition memory score (Kemp et al., 2017). To evaluate whether the DLB rat model established in this study also exhibited damaged recognition ability, object recognition test was conducted.

The object recognition test used rats’ nature of exploring objects. When a rat discovers a new object in a familiar environment, it tends to spend more time exploring the new object than exploring an object it is familiar with (Mumby et al., 2002). In the test, the rats needed to distinguish between the familiar and new objects. This required memory of the familiar object; that is, a rat’s preference for the new object indicated that it retained a memory of the familiar object (Grayson et al., 2015). Therefore, by analyzing the time spent by a rat to explore new and familiar objects, the ability of the rat to recognize objects was evaluated. In this study, the rats in the Sham+saline group spent more time exploring the new object, whereas no significant differences were observed in the time spent on exploring the new and old objects in the rats in the DLB+saline group, indicating the impaired ability of these rats to recognize objects. The rats in the DLB+CEF100 group spent higher amounts of time on exploring the new than on exploring old objects, indicating that CEF treatment repaired the object recognition ability of DLB rats. Similarly, we previously found that 5 days before the induction of PD, treatment with CEF (100 mg/kg/day) effectively prevented recognition impairment in a PD rat model (Hsu et al., 2015).

Learning is an ongoing experience-based process of behavior change and can be divided into non-associative learning and associative learning. Non-associative learning is a change in behavior response over time with a stimulus, including habituation and sensitivity. Associative learning is the course of behavior change with different stimuli that form links and is divided into classical conditioning and operational conditioning. If an individual gradually improves their accuracy or response speed through repeated exposure to a specific stimulus or procedure or if the individual is capable of learning motor or cognitive skills without the need to consciously recall past learning scenarios or rules for a task, the individual is defined as undergoing procedural learning (Cohen and Squire, 1980). Patients with DLB exhibit difficulties in initial learning and memory retrieval and are thus unable to acquire new knowledge or skills. Hamilton et al. used the California verbal learning test and Wechsler memory scale test to evaluate the learning and immediate recall of patients with DLB. The patients exhibited impairment in these two learning tasks, indicating difficulties in memory encoding (Hamilton et al., 2004). Moreover, one study demonstrated that despite intact declarative memory, skill learning (procedural learning) was poorer in patients with PD (Harrington et al., 1990). The active avoidance test in the present study used operational conditioning coupled with CS (lighting and tone) and UCS (electrical shock) to enable animals to learn the association between CS and UCS through negative reinforcement and execute avoidance responses when CS appeared. Hence, the behavioral data of the active avoidance test can be used to evaluate procedural learning ability (Lee et al., 2008). The results revealed that the avoidance response rate of the rats in the DLB+saline group was significantly lower than that of the rats in the Sham+saline group, indicating an impairment of procedural learning in DLB rats. Non-significant improvement of performance of DLB rats in the active avoidance test was observed after CEF treatment, which is sensitive to the mesolimbic and nigrostriatal dopamine (DA) level. However, the DA level in the striatum and SNc were actually significantly recovered by the current dose of CEF treatment while the motor impairment of DLB animals were found to be recovered also in the object recognition task. In this regard, we proposed that the current dosage of CEF may not be high enough for recovery of fear association memory in the DLB rats. CEF treatment prevented the learning deficit in DLB rats, indicating that CEF can be used to treat recognition impairment in DLB.

DAergic neurons in the nigrostriatal system is also observed in patients with DLB. Piggott et al. measured pathological changes in the putamen in patients with neurodegenerative diseases and found that DA concentration in the putamen was decreased by 72% in patients with DLB, decreased by 90% in patients with PD, and showed no change in patients with AD (Piggott et al., 1999). Moreover, the DA transporter level in the putamen was decreased by 57% in patients with DLB. Another study showed that 40–60% of the DAergic terminals in the striatum were damaged, and the substantial nigra showed moderate neuronal loss (Johnson et al., 2011). The striatum, a part of the basal ganglia, is involved in motion control, cognition, memory, and reward. Some neurodegenerative diseases, such as PD and Huntington’s disease, are related to the degeneration of DAergic terminals in the striatum. The present study discovered a substantial loss of DAergic terminals in the striatum of DLB rats, and the results were similar to those reported in a previous study (Johnson et al., 2011). However, after CEF treatment, the density of DAergic terminals in the striatum was effectively restored. The striatum is involved in the stimulus–response link (Colombo et al., 1989; Packard et al., 1989). Therefore, the striatum may be vital for the learning involved in the active avoidance test. To perform active avoidance in the test, the rats needed to recognize environmental stimuli (CS is the signal for initiating the avoidance response), identify changes in visual stimuli (two chambers in the shuttle box), and respond to a visuospatial short-term memory (remember the chamber from which it has recently left). As previously reported, low DA levels in the striatum interfere with the aforementioned functions (Cunha et al., 2001). A deficit of learning in the active avoidance test may be related to defects in the DAergic system, which was supported by the finding that CEF treatment, in addition to restoring the density of DAergic terminals in the striatum, improved the learning of DLB rats in the test.

The density of DAergic neurons in the SNc of DLB rats was decreased, but this was restored by CEF treatment. Regarding the clinical features of patients with DLB, in addition to cognitive dysfunction, motor impairment is observed. However, in the experiment, the DLB rats did not exhibit motor dysfunction (data not shown). Clinically, the “1-year rule” is used to distinguish DLB and PD dementia. If cognitive impairment occurs within 1 year following the onset of motor impairment or if cognitive impairment occurs earlier than motor impairment, DLB is diagnosed. Conversely, if cognitive impairment occurs 1 year after motor impairment occurs, PD with dementia (PDD) is diagnosed. Our present study demonstrated a cognitive deficit but not motor impairment in the DLB group. This may have been because of a short experimental period (only 28 days) and because the loss of DAergic neurons in the SNc (approximately 13%) was not sufficiently severe to cause motor impairment. By contrast, our previous study found that motor impairment was accompanied by more than 40% DAergic loss in the SNc in an MPTP-induced PD rat model (Weng et al., 2016). Notably, DA concentrations and DA transporter levels were decreased by 72 and 57%, respectively, in the putamen of aged (78 year-old) patients with DLB (Piggott et al., 1999). The density of DAergic neurons was effectively restored after CEF treatment in both the PD (Weng et al., 2016) and DLB rat models. Hence, based on the aforementioned results, we suggest that CEF may prevent DAergic degeneration in the nigrostriatal system in neurodegenerative disorders.

The hippocampus is involved in learning and memory. Using MRI, Chow et al. reported that compared with the normal group, patients with DLB exhibited significant atrophy in the hippocampal CA1 area (Chow et al., 2012). The loss of pyramidal neurons in the hippocampal CA1 and DG areas of rats in the DLB+saline group in the present study is consistent with the finding in DLB transgenic mice, which have exhibited abnormal α-syn accumulation and cell loss in the hippocampal CA1 and CA3 regions (Spencer et al., 2009; Lim et al., 2011; Overk et al., 2014). A post-mortem study showed abnormal α-syn accumulation in the hippocampal CA1 region of patients with DLB (Iseki et al., 2002). CEF treatment restored the density of pyramidal cells in the hippocampus, which may be attributed to the improvement in learning and memory in DLB rats.

The neuropathology of DLB is related to abnormal accumulation of α-syn in the cortex, which eventually forms Lewy bodies and Lewy neurites, causing neurotoxicity and neurodegeneration in the brain stem, cortex, and limbic system. In this study, α-syn gene vector was injected into the lateral ventricle of rats, which diffused because of the flow of the cerebrospinal fluid and facilitated the expression of α-syn throughout the regions of the brain to model the pathological features of DLB. High levels of α-syn accumulation were observed in the hippocampal DG area of the DLB+saline rats, and this accumulation may be involved in the lower density of pyramidal neurons in the hippocampus because abnormal accumulation of α-syn in the limbic system leads to cell loss and dysfunction of the hippocampus (Iseki et al., 2002). CEF has been reported to block the polymerization of α-syn and exert neuroprotective effects *in vitro* (Ruzza et al., 2014), which may be attributed to the effects of CEF in reducing the density of α-syn and restoring neuronal density in the hippocampus of DLB rats.

An adult animal’s brain retains its neurogenesis function, which repairs the nervous system for those with neurodegenerative diseases. Neurogenesis occurs mainly in the subventricular zone and the subgranular zone (SGZ). These two regions are known as neurogenic areas, and the other parts of the brain are known as non-neurogenic areas (Taupin, 2007), where in normal conditions, neurogenesis was though not to occur (Alvarez-Buylla and Garcıa-Verdugo, 2002; Seaberg and Van Der Kooy, 2002). This study demonstrated that CEF restored neuronal density in the hippocampus and substantia nigra of DLB rats. Hence, we further explored whether CEF promotes neurogenesis. BrdU is a synthetic nucleoside that replaces thymidine during DNA replication. Therefore, cells labeled with BrdU are newborn, and these cells can be used to evaluate the brain’s neurogenesis (Taupin, 2007). Because BrdU intercalates in any dividing or newborn cells. For more clearly analyzing neurogenesis in the future study, one should inject BrdU earlier or sacrifice rats later, for example, 3–4 weeks after BrdU injection, and perform double-staining with neuronal markers, for instance, a marker of immature neurons, doublecortin (DCX), or a marker of mature neurons, NeuN.

The hippocampus is a neurogenic area and is a crucial region responsible for learning and memory. Neurogenesis in the hippocampus has been demonstrated to be highly associated with hippocampus-dependent learning and memory. The promotion or inhibition of hippocampal neurogenesis may affect learning and memory. Gould et al. reported that hippocampus-dependent learning tasks increase the survival of newborn cells in the DG of rats (Gould et al., 1999). A study demonstrated that after placing rats in a rich environment for 4–8 weeks, hippocampal neurogenesis in the rats increased. In addition, the rats acquired more effective spatial learning skills in the Morris water maze test (Nilsson et al., 1999) and exhibited more efficient performance in inhibition of acoustic startle reflex (Iso et al., 2007). However, cytostatic drugs may suppress hippocampus-dependent learning (Shors et al., 2001). This indicates that newborn cells are involved in cognitive and memory functions, and the learning task also enhances neurogenesis in the hippocampus. A study demonstrated that neurogenic markers were abnormal in the hippocampus of patients with DLB (Johnson et al., 2011). Our previous studies discovered that CEF treatment reduced neuronal deficits in the nigrostriatal system and hippocampal CA1, CA3, and DG regions of the MPTP-induced PD rat model (Hsu et al., 2015). Additional studies have shown that CEF increases neurogenesis in the substantia nigra and hippocampal DG (Weng et al., 2016; Hsieh et al., 2017). Similarly, Zumkehr et al. (2015) reported a positive and significant correlation between CEF-induced increase of cognitive function and restoration of synaptic proteins in the hippocampal DG, CA1, and CA3 regions of a mice AD model, indicating a beneficial effect of CEF treatment in post-synaptic neurons. Because the SGZ is the main region in which neurogenesis occurs, it is speculated that CEF repairs neuronal deficits in the hippocampal DG by increasing neurogenesis (Hsu et al., 2015; Weng et al., 2016). The present study demonstrated that the number of newborn cells in the hippocampal DG of rats in the DLB+saline group was significantly lower than that of rats in the Sham+saline group. CEF treatment restored the aforementioned deficits. To our knowledge, this is the first study demonstrating that CEF prevents loss of neurogenesis in the brain of DLB rats.

The substantia nigra was previously believed to be part of the non-neurogenic area, not producing new neurons under normal conditions (Alvarez-Buylla and Garcıa-Verdugo, 2002; Seaberg and Van Der Kooy, 2002). However, one study found new neurons in the pars reticulata of the substantia nigra (SNr) of patients with PD (Yoshimi et al., 2005). Similarly, neurogenesis was observed in PD rats and primate animal models (Lie et al., 2002; Yoshimi et al., 2005). These results support our previous finding that compared with the controls, PD rats had fewer newborn cells in the SNr. CEF treatment restored this deficit (Hsieh et al., 2017). Similarly, the present study discovered fewer newborn cells in the SNr of DLB rats, but the number of cells was restored by CEF treatment. Hence, we suggest that the recovery of neuronal density in the substantia nigra may have been due to increased neurogenesis. Because mature DAergic cells are mostly located in the SNc and we did not observe numerous newborn cells there, we can infer that SNc may not have been the main region in which new cells were produced. This study found that the number of newborn cells in the SNr was significantly higher than that in the SNc (approx. 130 cells vs. 30 cells). Thus, SNr may play a role in producing and incubating new cells. By increasing the number of newborn cells in the SNr and enabling the gradual migration of these cells to the SNc, CEF prevented the neuronal deficit in the SNc. CEF may thus be beneficial for the treatment of neurodegenerative diseases.

Our recent study, injecting rAAV into the ventricle and Aβ into the hippocampus, revealed that Aβ inhibits autophagy, increases intracellular ROS accumulation and mitochondrial dysfunction, and enhances α-syn aggregation in α-syn-overexpressed neurons (Lin et al., 2016). The method used in the present study for inducing pathological changes resulted in neurodegeneration and behavioral deficits similar to that in DLB. Analyzing the relationship between the level of Aβ and α-syn will be necessary in the future study for interpretation of the data and the validity of the model. Furthermore, in the future study, when establishing DLB or another synucleinopathy model, a better method should be designed to allow Aβ and α-syn to be properly expressed in the appropriate brain regions.

There are some literatures including our own research reporting the effects of CEF in normal rats. A dose-dependent effect of CEF on GLT-1 expression was observed on the astrocytes in the striatum and hippocampal CA1 in not only PD rats but also in normal rats. But CEF has no significant effect on behavior in normal rats, which may because of ceiling effect (Hsu et al., 2015). In view of this, no Sham+CEF group was assigned in the present study. Using fluorescent staining and high-resolution camera in the future study will be helpful for producing clear image and for analyzing changes in the neuronal tissue. Phospho-specific staining will be needed for an appropriate presentation of α-syn because α-syn is ubiquitously expressed.

## Conclusion

In conclusion, CEF treatment repaired deficits of pyramidal neurons in the hippocampus of DLB rats and improved the density of DAergic neurons in the nigrostriatal system. Furthermore, neurogenesis in the DG and SNr was enhanced by CEF treatment. The results of behavioral tests revealed that CEF treatment improved learning and cognition in DLB rats. Thus, CEF may have potential for treating DLB.

## Ethics Statement

All experimental procedures were performed according to the NIH Guide for the Care and Use of Laboratory Animals and were approved by the Animal Care Committee of Chung Shan Medical University (IACUC approval No. 1455). All efforts were made to minimize animal suffering and to reduce the number of animals used (Castelhano-Carlos and Baumans, 2009).

## Author Contributions

All the authors contributed to the experiment. Y-JH conceived and performed the study, and wrote the manuscript for publication. C-LL and C-SH critically reviewed the manuscript.

## Conflict of Interest Statement

The authors declare that the research was conducted in the absence of any commercial or financial relationships that could be construed as a potential conflict of interest.

## References

[B1] Aarsland D. Ballard C. Walker Z. Bostrom F. Alves G. Kossakowski K. (2009). Memantine in patients with Parkinson’s disease dementia or dementia with Lewy bodies: a double-blind, placebo-controlled, multicentre trial. *Lancet Neurol.* 8 613–618. 10.1016/S1474-4422(09)70146-2 19520613

[B2] Aldrin-Kirk P. Davidsson M. Holmqvist S. Li J. Y. Bjorklund T. (2014). Novel AAV-based rat model of forebrain synucleinopathy shows extensive pathologies and progressive loss of cholinergic interneurons. *PLoS One* 9:e100869. 10.1371/journal.pone.0100869 24999658 PMC4085060

[B3] Alvarez-Buylla A. Garcıa-Verdugo J. M. (2002). Neurogenesis in adult subventricular zone. *J. Neurosci.* 22 629–634. 11826091 10.1523/JNEUROSCI.22-03-00629.2002 PMC6758521

[B4] Armstrong R. A. Cairns N. J. (2009). Size frequency distribution of the beta-amyloid (abeta) deposits in dementia with Lewy bodies with associated Alzheimer’s disease pathology. *Neurol. Sci.* 30 471–477. 10.1007/s10072-009-0135-6 19768369 PMC2809796

[B5] Beyer K. Domingo-Sabat M. Ariza A. (2009). Molecular pathology of Lewy body diseases. *Int. J. Mol. Sci.* 10 724–745. 10.3390/ijms10030724 19399218 PMC2671999

[B6] Burre J. Sharma M. Sudhof T. C. (2015). Definition of a molecular pathway mediating alpha-synuclein neurotoxicity. *J. Neurosci.* 35 5221–5232. 10.1523/JNEUROSCI.4650-14.2015 25834048 PMC4380997

[B7] Campbell B. C. Li Q.-X. Culvenor J. G. Jäkälä P. Cappai R. Beyreuther K. (2000). Accumulation of insoluble α-synuclein in dementia with Lewy bodies. *Neurobiol. Dis.* 7 192–200. 10860784 10.1006/nbdi.2000.0286

[B8] Castelhano-Carlos M. J. Baumans V. (2009). The impact of light, noise, cage cleaning and in-house transport on welfare and stress of laboratory rats. *Lab. Anim.* 43 311–327. 10.1258/la.2009.0080098 19505937

[B9] Chang C. C. Li H. H. Chang Y. T. Ho Y. J. Hsieh L. J. Chiu P. Y. (2018). Abeta exacerbates alpha-synuclein-induced neurotoxicity through impaired insulin signaling in alpha-synuclein-overexpressed human SK-N-MC neuronal cells. *CNS Neurosci. Ther.* 24 47–57. 10.1111/cns.12772 29092095 PMC6489723

[B10] Chesselet M. F. (2008). In vivo alpha-synuclein overexpression in rodents a useful model of Parkinson’s disease. *Exp. Neurol.* 209 22–27. 17949715 10.1016/j.expneurol.2007.08.006 PMC2262051

[B11] Chow N. Aarsland D. Honarpisheh H. Beyer M. K. Somme J. H. Elashoff D. (2012). Comparing hippocampal atrophy in Alzheimer’s dementia and dementia with lewy bodies. *Dement. Geriatr. Cogn. Disord.* 34 44–50. 10.1159/000339727 22922563 PMC3470878

[B12] Churchyard A. Lees A. J. (1997). The relationship between dementia and direct involvement of the hippocampus and amygdala in Parkinson’s disease. *Neurology* 49 1570–1576. 9409348 10.1212/wnl.49.6.1570

[B13] Cohen N. J. Squire L. R. (1980). Preserved learning and retention of pattern-analyzing skill in amnesia: dissociation of knowing how and knowing that. *Science* 210 207–210. 7414331 10.1126/science.7414331

[B14] Colombo P. J. Davis H. P. Volpe B. T. (1989). Allocentric spatial and tactile memory impairments in rats with dorsal caudate lesions are affected by preoperative behavioral training. *Behav. Neurosci.* 103:1242. 2610917 10.1037//0735-7044.103.6.1242

[B15] Cunha C. D. Gevaerd M. S. Vital M. A. Miyoshi E. Andreatini R. Silveira R. (2001). Memory disruption in rats with nigral lesions induced by MPTP a model for early Parkinson’s disease amnesia. *Behav. Brain Res.* 124 9–18. 11423161 10.1016/s0166-4328(01)00211-x

[B16] Galvin J. E. Uryu K. Lee V. M. Trojanowski J. Q. (1999). Axon pathology in Parkinson’s disease and Lewy body dementia hippocampus contains alpha-, beta-, and gamma-synuclein. *Proc. Natl. Acad. Sci. U.S.A.* 96 13450–13455. 10557341 10.1073/pnas.96.23.13450 PMC23968

[B17] Gould E. Beylin A. Tanapat P. Reeves A. Shors T. J. (1999). Learning enhances adult neurogenesis in the hippocampal formation. *Nat. Neurosci.* 2 260–265. 10195219 10.1038/6365

[B18] Grayson B. Leger M. Piercy C. Adamson L. Harte M. Neill J. C. (2015). Assessment of disease-related cognitive impairments using the novel object recognition (NOR) task in rodents. *Behav. Brain Res.* 285 176–193. 10.1016/j.bbr.2014.10.025 25447293

[B19] Hamilton J. M. Salmon D. P. Galasko D. Delis D. C. Hansen L. A. Masliah E. (2004). A comparison of episodic memory deficits in neuropathologically-confirmed Dementia with Lewy bodies and Alzheimer’s disease. *J. Int. Neuropsychol. Soc.* 10 689–697. 15327716 10.1017/S1355617704105043

[B20] Harrington D. L. Haaland K. Y. Yeo R. A. Marder E. (1990). Procedural memory in Parkinson’s disease: impaired motor but not visuoperceptual learning. *J. Clin. Exp. Neuropsychol.* 12 323–339. 2341560 10.1080/01688639008400978

[B21] Hishikawa N. Hashizume Y. Yoshida M. Sobue G. (2003). Clinical and neuropathological correlates of Lewy body disease. *Acta Neuropathol.* 105 341–350. 12624787 10.1007/s00401-002-0651-4

[B22] Ho Y. J. Weng J. C. Lin C. L. Shen M. S. Li H. H. Liao W. C. (2018). Ceftriaxone treatment for neuronal deficits: a histological and MEMRI study in a rat model of dementia with Lewy bodies. *Behav. Neurol.* 2018:4618716. 10.1155/2018/4618716 30154934 PMC6092970

[B23] Hsieh M. H. Gu S. L. Ho S. C. Pawlak C. R. Lin C. L. Ho Y. J. (2012a). Effects of MK-801 on recognition and neurodegeneration in an MPTP-induced Parkinson’s rat model. *Behav. Brain Res.* 229 41–47. 10.1016/j.bbr.2011.12.035 22227506

[B24] Hsieh M. H. Ho S. C. Yeh K. Y. Pawlak C. R. Chang H. M. Ho Y. J. (2012b). Blockade of metabotropic glutamate receptors inhibits cognition and neurodegeneration in an MPTP-induced Parkinson’s disease rat model. *Pharmacol. Biochem. Behav.* 102 64–71. 10.1016/j.pbb.2012.03.022 22487770

[B25] Hsieh M. H. Meng W. Y. Liao W. C. Weng J. C. Li H. H. Su H. L. (2017). Ceftriaxone reverses deficits of behavior and neurogenesis in an MPTP-induced rat model of Parkinson’s disease dementia. *Brain Res. Bull.* 132 129–138. 10.1016/j.brainresbull.2017.05.015 28576659

[B26] Hsu C. Y. Hung C. S. Chang H. M. Liao W. C. Ho S. C. Ho Y. J. (2015). Ceftriaxone prevents and reverses behavioral and neuronal deficits in an MPTP-induced animal model of Parkinson’s disease dementia. *Neuropharmacology* 91 43–56. 10.1016/j.neuropharm.2014.11.023 25499022

[B27] Huang C. K. Chang Y. T. Amstislavskaya T. G. Tikhonova M. A. Lin C. L. Hung C. S. (2015). Synergistic effects of ceftriaxone and erythropoietin on neuronal and behavioral deficits in an MPTP-induced animal model of Parkinson’s disease dementia. *Behav. Brain Res.* 294 198–207. 10.1016/j.bbr.2015.08.011 26296668

[B28] Ingelsson M. (2016). Alpha-synuclein oligomers-neurotoxic molecules in Parkinson’s disease and other Lewy body disorders. *Front. Neurosci.* 10:408 10.3389/fnins.2016.00408 PMC5011129 27656123

[B29] Iseki E. Takayama N. Marui W. Uéda K. Kosaka K. (2002). Relationship in the formation process between neurofibrillary tangles and Lewy bodies in the hippocampus of dementia with Lewy bodies brains. *J. Neurol. Sci.* 195 85–91. 11867079 10.1016/s0022-510x(01)00689-x

[B30] Iso H. Simoda S. Matsuyama T. (2007). Environmental change during postnatal development alters behaviour, cognitions and neurogenesis of mice. *Behav. Brain Res.* 179 90–98. 17321608 10.1016/j.bbr.2007.01.025

[B31] Johnson M. Ekonomou A. Hobbs C. Ballard C. G. Perry R. H. Perry E. K. (2011). Neurogenic marker abnormalities in the hippocampus in dementia with Lewy bodies. *Hippocampus* 21 1126–1136. 10.1002/hipo.20826 20665591

[B32] Kemp J. Philippi N. Phillipps C. Demuynck C. Albasser T. Martin-Hunyadi C. (2017). Cognitive profile in prodromal dementia with Lewy bodies. *Alzheimers Res. Ther.* 9:19. 10.1186/s13195-017-0242-1 28302161 PMC5356316

[B33] Kim W. S. Kagedal K. Halliday G. M. (2014). Alpha-synuclein biology in Lewy body diseases. *Alzheimers Res. Ther.* 6:73. 10.1186/s13195-014-0073-2 25580161 PMC4288216

[B34] Ko L. W. Ko H. H. Lin W. L. Kulathingal J. G. Yen S. H. (2008). Aggregates assembled from overexpression of wild-type alpha-synuclein are not toxic to human neuronal cells. *J. Neuropathol. Exp. Neurol.* 67 1084–1096. 10.1097/NEN.0b013e31818c3618 18957893 PMC2768257

[B35] Kornelius E. Lin C. L. Chang H. H. Li H. H. Huang W. N. Yang Y. S. (2015). DPP-4 Inhibitor linagliptin attenuates abeta-induced cytotoxicity through activation of AMPK in neuronal cells. *CNS Neurosci. Ther.* 21 549–557. 10.1111/cns.12404 26010513 PMC5033019

[B36] Kosaka K. Oyanagi S. Matsushita M. Hori A. (1976). Presenile dementia with Alzheimer-, Pick- and Lewy-body changes. *Acta Neuropathol.* 36 221–233. 188300 10.1007/BF00685366

[B37] Kronenberg G. Reuter K. Steiner B. Brandt M. D. Jessberger S. Yamaguchi M. (2003). Subpopulations of proliferating cells of the adult hippocampus respond differently to physiologic neurogenic stimuli. *J. Comp. Neurol.* 467 455–463. 14624480 10.1002/cne.10945

[B38] Lee Y. T. Wang W. F. Cheng C. W. Wu S. L. Pawlak C. R. Ho Y. J. (2008). Effects of escapable and inescapable stressors on behavior and interleukin-2 in the brain. *Neuroreport* 19 1243–1247. 10.1097/WNR.0b013e32830b5d86 18628674

[B39] Lie D. C. Dziewczapolski G. Willhoite A. R. Kaspar B. K. Shults C. W. Gage F. H. (2002). The adult substantia nigra contains progenitor cells with neurogenic potential. *J. Neurosci.* 22 6639–6649. 12151543 10.1523/JNEUROSCI.22-15-06639.2002 PMC6758128

[B40] Lim Y. Kehm V. M. Lee E. B. Soper J. H. Li C. Trojanowski J. Q. (2011). alpha-Syn suppression reverses synaptic and memory defects in a mouse model of dementia with Lewy bodies. *J. Neurosci.* 31 10076–10087. 10.1523/JNEUROSCI.0618-11.2011 21734300 PMC3144489

[B41] Lin C. L. Cheng Y. S. Li H. H. Chiu P. Y. Chang Y. T. Ho Y. J. (2016). Amyloid-beta suppresses AMP-activated protein kinase (AMPK) signaling and contributes to alpha-synuclein-induced cytotoxicity. *Exp. Neurol.* 275 84–98. 10.1016/j.expneurol.2015.10.009 26515689

[B42] Macijauskienë J. Lesauskaitë V. (2012). Dementia with lewy bodies: the principles of diagnostics, treatment, and management. *Medicina* 48 1–8. 22481369

[B43] Marsh S. E. Blurton-Jones M. (2012). Examining the mechanisms that link beta-amyloid and alpha-synuclein pathologies. *Alzheimers Res. Ther.* 4:11. 10.1186/alzrt109 22546279 PMC4054672

[B44] McKeith I. G. Dickson D. W. Lowe J. Emre M. O’Brien J. T. Feldman H. (2005). Diagnosis and management of dementia with Lewy bodies: third report of the DLB Consortium. *Neurology* 65 1863–1872. 16237129 10.1212/01.wnl.0000187889.17253.b1

[B45] Mochizuki H. Yamada M. Mizuno Y. (2006). α-synuclein overexpression model. *J. Neural Transm. Suppl.* 70 281–284. 17017543

[B46] Mosimann U. P. Mather G. Wesnes K. A. O’Brien J. T. Burn D. J. McKeith I. G. (2004). Visual perception in parkinson disease dementia and dementia with Lewy bodies. *Neurology* 63 2091–2096. 15596755 10.1212/01.wnl.0000145764.70698.4e

[B47] Mueller C. Ballard C. Corbett A. Aarsland D. (2017). The prognosis of dementia with Lewy bodies. *Lancet Neurol.* 16 390–398. 10.1016/S1474-4422(17)30074-1 28342649

[B48] Mumby D. G. Gaskin S. Glenn M. J. Schramek T. E. Lehmann H. (2002). Hippocampal damage and exploratory preferences in rats: memory for objects, places, and contexts. *Learn. Mem.* 9 49–57. 11992015 10.1101/lm.41302 PMC155935

[B49] Nilsson M. Perfilieva E. Johansson U. Orwar O. Eriksson P. S. (1999). Enriched environment increases neurogenesis in the adult rat dentate gyrus and improves spatial memory. *J. Neurobiol.* 39 569–578. 10380078 10.1002/(sici)1097-4695(19990615)39:4<569::aid-neu10>3.0.co;2-f

[B50] Norman K. A. O’reilly R. C. (2003). Modeling hippocampal and neocortical contributions to recognition memory a complementary-learning-systems approach. *Psychol. Rev.* 110 611–646. 14599236 10.1037/0033-295X.110.4.611

[B51] Oda H. Yamamoto Y. Maeda K. (2009). Neuropsychological profile of dementia with Lewy bodies. *Psychogeriatrics* 9 85–90. 10.1111/j.1479-8301.2009.00283.x 19604331

[B52] Ouyang Y. B. Voloboueva L. A. Xu L. J. Giffard R. G. (2007). Selective dysfunction of hippocampal CA1 astrocytes contributes to delayed neuronal damage after transient forebrain ischemia. *J. Neurosci.* 27 4253–4260. 17442809 10.1523/JNEUROSCI.0211-07.2007 PMC3140959

[B53] Overk C. R. Cartier A. Shaked G. Rockenstein E. Ubhi K. Spencer B. (2014). Hippocampal neuronal cells that accumulate alpha-synuclein fragments are more vulnerable to Abeta oligomer toxicity via mGluR5–implications for dementia with Lewy bodies. *Mol. Neurodegener.* 9:18. 10.1186/1750-1326-9-18 24885390 PMC4041038

[B54] Packard M. G. Hirsh R. White N. M. (1989). Differential effects of fornix and caudate nucleus lesions on two radial maze tasks: evidence for multiple memory systems. *J. Neurosci.* 9 1465–1472. 2723738 10.1523/JNEUROSCI.09-05-01465.1989 PMC6569845

[B55] Paxinos G. Watson C. (1986). *The rat Brain in Stereotaxic Coordinates.* London: Academic Press.

[B56] Piggott M. A. Marshall E. F. Thomas N. Lloyd S. Court J. A. Jaros E. (1999). Striatal dopaminergic markers in dementia with Lewy bodies, Alzheimer’s and Parkinson’s diseases: rostrocaudal distribution. *Brain* 122( Pt 8), 1449–1468. 10430831 10.1093/brain/122.8.1449

[B57] Rothstein J. D. Patel S. Regan M. R. Haenggeli C. Huang Y. H. Bergles D. E. (2005). β-Lactam antibiotics offer neuroprotection by increasing glutamate transporter expression. *Nature* 433 73–77. 15635412 10.1038/nature03180

[B58] Ruzza P. Siligardi G. Hussain R. Marchiani A. Islami M. Bubacco L. (2014). Ceftriaxone blocks the polymerization of α-synuclein and exerts neuroprotective effects in vitro. *ACS Chem. Neurosci.* 5 30–38. 10.1021/cn400149k 24099687 PMC3894726

[B59] Samuel W. Alford M. Hofstetter C. R. Hansen L. (1997). Dementia with Lewy bodies versus pure Alzheimer disease: differences in cognition, neuropathology, cholinergic dysfunction, and synapse density. *J. Neuropathol. Exp. Neurol.* 56 499–508. 9143263 10.1097/00005072-199705000-00006

[B60] Schulz-Schaeffer W. J. (2010). The synaptic pathology of alpha-synuclein aggregation in dementia with Lewy bodies, Parkinson’s disease and Parkinson’s disease dementia. *Acta Neuropathol.* 120 131–143. 10.1007/s00401-010-0711-0 20563819 PMC2892607

[B61] Seaberg R. M. Van Der Kooy D. (2002). Adult rodent neurogenic regions: the ventricular subependyma contains neural stem cells, but the dentate gyrus contains restricted progenitors. *J. Neurosci.* 22 1784–1793. 11880507 10.1523/JNEUROSCI.22-05-01784.2002 PMC6758891

[B62] Shors T. J. Miesegaes G. Beylin A. Zhao M. Rydel T. Gould E. (2001). Neurogenesis in the adult is involved in the formation of trace memories. *Nature* 410 372–376. 11268214 10.1038/35066584

[B63] Singleton A. B. Farrer M. Johnson J. Singleton A. Hague S. Kachergus J. (2003). alpha-Synuclein locus triplication causes Parkinson’s disease. *Science* 302:841. 10.1126/science.1090278 14593171

[B64] Spencer B. Potkar R. Trejo M. Rockenstein E. Patrick C. Gindi R. (2009). Beclin 1 gene transfer activates autophagy and ameliorates the neurodegenerative pathology in alpha-synuclein models of Parkinson’s and Lewy body diseases. *J. Neurosci.* 29 13578–13588. 10.1523/JNEUROSCI.4390-09.2009 19864570 PMC2812014

[B65] Spillantini M. G. Crowther R. A. Jakes R. Hasegawa M. Goedert M. (1998). a-Synuclein in filamentous inclusions of Lewy bodies from Parkinson’s disease and dementia with Lewy bodies. *Proc. Natl. Acad. Sci. U.S.A.* 95 6469–6473. 9600990 10.1073/pnas.95.11.6469 PMC27806

[B66] Spillantini M. G. Schmidt M. L. Lee V. M. Trojanowski J. Q. Jakes R. Goedert M. (1997). Alpha-synuclein in Lewy bodies. *Nature* 388 839–840. 9278044 10.1038/42166

[B67] Tagliafierro L. Chiba-Falek O. (2016). Up-regulation of SNCA gene expression: implications to synucleinopathies. *Neurogenetics* 17 145–157. 10.1007/s10048-016-0478-0 26948950 PMC4907864

[B68] Taupin P. (2007). BrdU immunohistochemistry for studying adult neurogenesis: paradigms, pitfalls, limitations, and validation. *Brain Res. Rev.* 53 198–214. 17020783 10.1016/j.brainresrev.2006.08.002

[B69] Valdinocci D. Radford R. A. Siow S. M. Chung R. S. Pountney D. L. (2017). Potential modes of intercellular alpha-synuclein transmission. *Int. J. Mol. Sci.* 18:469. 10.3390/ijms18020469 28241427 PMC5344001

[B70] van Marum R. J. (2009). Update on the use of memantine in Alzheimer’s disease. *Neuropsychiatr. Dis. Treat.* 5 237–247. 19557118 10.2147/ndt.s4048 PMC2695219

[B71] Wang H. F. Yu J. T. Tang S. W. Jiang T. Tan C. C. Meng X. F. (2015). Efficacy and safety of cholinesterase inhibitors and memantine in cognitive impairment in Parkinson’s disease, Parkinson’s disease dementia, and dementia with Lewy bodies: systematic review with meta-analysis and trial sequential analysis. *J. Neurol. Neurosurg. Psychiatry* 86 135–143. 10.1136/jnnp-2014-307659 24828899

[B72] Weil R. S. Lashley T. L. Bras J. Schrag A. E. Schott J. M. (2017). Current concepts and controversies in the pathogenesis of Parkinson’s disease dementia and Dementia with Lewy Bodies. *F1000Res* 6:1604. 10.12688/f1000research.11725.1 28928962 PMC5580419

[B73] Weng J. C. Tikhonova M. A. Chen J. H. Shen M. S. Meng W. Y. Chang Y. T. (2016). Ceftriaxone prevents the neurodegeneration and decreased neurogenesis seen in a Parkinson’s disease rat model: an immunohistochemical and MRI study. *Behav. Brain Res.* 305 126–139. 10.1016/j.bbr.2016.02.034 26940602

[B74] Yoshimi K. Ren Y. R. Seki T. Yamada M. Ooizumi H. Onodera M. (2005). Possibility for neurogenesis in substantia nigra of parkinsonian brain. *Ann. Neurol.* 58 31–40. 15912513 10.1002/ana.20506

[B75] Zaccai J. McCracken C. Brayne C. (2005). A systematic review of prevalence and incidence studies of dementia with Lewy bodies. *Age Ageing* 34 561–566. 16267179 10.1093/ageing/afi190

[B76] Zarranz J. J. Alegre J. Gomez-Esteban J. C. Lezcano E. Ros R. Ampuero I. (2004). The new mutation, E46K, of alpha-synuclein causes Parkinson and Lewy body dementia. *Ann. Neurol.* 55 164–173. 14755719 10.1002/ana.10795

[B77] Zhang L. Yan R. Zhang Q. Wang H. Kang X. Li J. (2013). Survivin, a key component of the Wnt/β-catenin signaling pathway, contributes to traumatic brain injury-induced adult neurogenesis in the mouse dentate gyrus. *Int. J. Mol. Med.* 32 867–875. 10.3892/ijmm.2013.1456 23900556

[B78] Zumkehr J. Rodriguez-Ortiz C. J. Cheng D. Kieu Z. Wai T. Hawkins C. (2015). Ceftriaxone ameliorates tau pathology and cognitive decline via restoration of glial glutamate transporter in a mouse model of Alzheimer’s disease. *Neurobiol. Aging* 36 2260–2271. 10.1016/j.neurobiolaging.2015.04.005 25964214

